# Anaphylaxis after SonoVue: A Case Report and a Literature Review

**DOI:** 10.3390/jcm13175018

**Published:** 2024-08-24

**Authors:** David Longhino, Alessandro Buonomo, Maria Assunta Zocco, Maria Elena Ainora, Giorgio Esposto, Irene Mignini, Lucia Cerrito, Francesca Romana Ponziani, Antonio Gasbarrini, Eleonora Nucera, Arianna Aruanno

**Affiliations:** 1 Allergy Unit, Fondazione Policlinico Universitario “A. Gemelli” IRCCS, Catholic University of Rome, 00136 Roma, Italy; david.longhino@gmail.com (D.L.); alessandro.buonomo@policlinicogemelli.it (A.B.); eleonora.nucera@policlinicogemelli.it (E.N.); 2 CEMAD Digestive Disease Center, Fondazione Policlinico Universitario “A. Gemelli” IRCCS, Catholic University of Rome, 00136 Roma, Italy; mariaassunta.zocco@policlinicogemelli.it (M.A.Z.); mariaelena.ainora@policlinicogemelli.it (M.E.A.); giorgio.esposto01@icatt.it (G.E.); irene.mignini@policlinicogemelli.it (I.M.); lucia.cerrito@guest.policlinicogemelli.it (L.C.); francescaromana.ponziani@policlinicogemelli.it (F.R.P.); antonio.gasbarrini@policlinicogemelli.it (A.G.)

**Keywords:** sonoVue, ecography, anaphylaxis

## Abstract

SonoVue (Bracco, Milan, Italy) is a drug used in ultrasonography for the purpose of increasing the echogenicity of blood or fluids by improving the signal-to-noise ratio. **Background/Objectives/Methods**: We described a case of anaphylaxis due to SonoVue and performed a literature review. **Results and Conclusions**: We reported a case of anaphylaxis secondary to the administration of SonoVue and described all the 13 literature cases. Given its widespread use and the potentially dangerous nature of the reactions it can cause, it is advisable to know how to promptly recognize fatal reactions.

## 1. Introduction

Sulphur hexafluoride (SonoVue—Bracco, Milan, Italy) is a contrast agent used in ultrasonography for the purpose of increasing the echogenicity of blood or fluids, thereby improving the signal-to-noise ratio in imaging. It is mainly utilized in echocardiography to study the heart and great vessels, but also finds applications in studying microcirculation, such as in breast and liver lesions, as well as the urinary excretory tract. The active ingredient in SonoVue is sulfur hexafluoride (SF6), a high-intensity, non-organic, colorless, odorless, tasteless and inert gas. This gas is injected intravenously in the form of “microbubbles”, which are produced by mixing a powder (solute) containing SF6, macrogol 4000, distearoylphosphatidylcholine, dipalmitoylphosphatidylglycerol sodium and palmitic acid with sodium chloride 0.9% (solvent).

The use of SonoVue is contraindicated in patients with hypersensitivity to the active ingredient or to any of its excipient, presence of left-right shunts, severe pulmonary hypertension, uncontrolled systemic hypertension, adult respiratory distress syndrome. Additionally, it should not be administered in combination with dobutamine in patients with a clinical picture of suspected cardiovascular instability in whom dobutamine is contraindicated [1].

The most common adverse reactions reported are: headache, nausea, abdominal pain, skin rash, injection site reactions, hypotension, pruritus, hypersensitivity to sulfur hexafluoride or any of the excipients. Although there are no definite data from the European Medicines Agency (EMA) regarding the risk of myocardial infarction, myocardial ischemia, or Kounis syndrome (allergic acute coronary syndrome), these concerns remain under investigation [2,3,4,5].

In this article, we present one case of anaphylaxis occurring after SonoVue infusion and review the other 13 cases of hypersensitivity reactions reported in the literature after SonoVue and Lumasone (which contains exactly the same molecules of SonoVue) infusions.

## 2. Methods

Allergological evaluation in the above patient was performed using skin prick (SPTs) and intradermal tests (IDTs), according to EAACI guidelines within 6 months from the reaction [6]. A maximum concentration up to the undiluted solution was used for all tests. In addition, ten healthy individuals were enrolled as controls with negative results at the concentration above mentioned.

In order to describe the reported cases of anaphylaxis due to SonoVue, a literature review was carried out on Pubmed (MEDLINE) from inception to 31 April 2024, using the following MeSH headings and keywords: “anaphylaxis AND sulfur hexafluoride”, “anaphylaxis AND SonoVue”, “anaphylaxis AND Lumason”. We also selected cases where Lumason was used to create more robust evidence.

We found 13 described cases of anaphylaxis after sulfur hexafluoride in 11 articles; the 13 cases are described in Table 1.

## 3. Case Report

We describe the case of a 67-year-old male who underwent ultrasonographic examination after intravenous infusion of SonoVue to investigate liver lesions (pre-infusion of chemotherapy). His medical history included arterial hypertension and alcohol-related liver cirrhosis (Child–Pugh A6) complicated by portal hypertension and hepatocarcinoma; in 2018, he underwent liver resection (segments IVa, VII, VIII) and subsequently transhepatic radioembolization (2022) to segments IVb-V. Risk factors for anaphylaxis (e.g., clonal mast cell disease, hereditary alpha-tryptasemia) were absent. His home therapy consisted of carvedilol 12.5 mg, esomeprazole 40 mg, perindopril 5 mg, indapamide 1.25 and amlodipine 5 mg.

Approximately three minutes after SonoVue infusion, the patient presented with pruritus, dyspnea, bilateral expiratory wheezing, cyanosis of the extremities and cardiovascular arrest, for which he was given methylprednisolone 80 mg iv, chlorphenamine 10 mg iv, and then a cardiopulmonary resuscitation, laryngeal mask placement and midazolam infusion were performed. Unfortunately, serum tryptase assay could not be performed within 4 h of the reaction due to its unavailability. Basal serum tryptase carried out 4 days after the reaction was 2.7 mcg/L.

About twenty days earlier the patient underwent the same procedure without presenting any reaction.

Sixteen weeks after the reaction, the patient was referred to Our Center to investigate a possible allergic etiopathogenesis to the reaction presented after the infusion of SonoVue. After signing the informed consent, skin prick tests and intradermal reactions with SonoVue (1:10,000, 1:1000, 1:100, 1:10 and pure solutions, in accordance with a previous case report published by Levano et al.) [10] were performed on the volar face of the forearms and were negative. A basophil activation test (BAT, Flow-Cast, Bühlmann Laboratories AG, Schönenbuch, Switzerland) for SonoVue was carried out and came back negative; BAT was carried out using the same concentrations as the skin tests and a Basophil activation > 5% and a stimulation index > 2 were considered a positive result.

A challenge was not performed due to the reaction presented by the patient.

## 4. Discussion

Anaphylaxis is a severe, systemic, potentially life-threatening syndrome. The diagnosis is clinical, but as suggested in the latest EAACI guidelines, measuring serum tryptase one-half to two hours after the onset of the reaction, and baseline serum tryptase at least 24 h after complete resolution of symptoms, although will not help to make a diagnosis in an emergency setting, may be helpful in the subsequent allergy consultation [18].

Although extremely rare, hypersensitivity reactions due to sulphur hexafluoride are described in the literature.

Shang et al., in a meta-analysis conducted on 463434 procedures using sulfur hexafluoride microbubbles in China, reported a frequency of mild allergic reactions (defined by the authors as limited urticaria, skin oedema, scratchy throat) of 0.0026%, moderate allergic reactions (generalized urticaria, facial oedema, throat tightness without dyspnea, wheezing/bronchospasm without hypoxia) of 0.0047% and severe allergic reactions (anaphylactic shock with cardiovascular involvement, wheezing/bronchospasm with hypoxia) of 0.0030% [19].

Geleijnse et al. evaluated 352 cardiac patients during a 4-year period. Seven patients (2.0%) experienced adverse effects; of these, four patients (1.1%) had mild allergic reactions causing skin erythema and mild sinus tachycardia, and three patients (0.9%) experienced a severe allergic reaction resulting in (nonfatal) shock [8]. In the majority of cases, contrast media were used in a cardiovascular context, but Solivetti et al., Levano et al. and Olson et al. described three cases unrelated to heart disease [10,11,15].

Only Levano et al. and Coudray et al. carried out an allergy skin test assessment. In their case report, Coudray et al. performed an allergy evaluation for SonoVue (not reporting dilutions) and PEG with a negative result, while the serum tryptase assay performed during the reaction was positive (147 ugL/L). Levano et al. carried out an allergy test for SonoVue (starting, as in our case, from a dilution of 1:10,000 to a pure concentration) and a serum tryptase assay, both carried out several months after the reaction and both with negative results [7,10].

Steinhauer et al. described a case of anaphylaxis with Kounis syndrome in a female patient after the first exposure of sulfur hexafluoride ultrasound enhancing agent, which has been attributed to a complement activation-related pseudoallergy (CARPA) [13].

Of the four cases described in the literature in which Lumason was used [14,15,16,17], unfortunately two of them could not be accessed in full. Interestingly, Olson et al. described a case in a patient suffering from mastocytosis [15].

The negative allergological evaluations performed in the above-mentioned case reports and in our case could be explained by a non-IgE-mediated mechanism underlying the reactions reported. In fact, the pathogenesis of anaphylaxis can be distinguished, according to the underlying mechanism, into immunological (classified into an IgE-dependent and an IgE-independent form), non-immunological (due to direct mast cell activation and/or complement), and idiopathic (in which no trigger is apparently present) [20]. In four cases [7,13,15,17], an increase in serum tryptase has been documented; this finding is very interesting because it emphasizes the involvement of the immune system in these reactions, although this marker dosage must be carried out within a certain time after the reaction [21]. As already mentioned, unfortunately no serum tryptase assay was performed during the reaction; its measurement would certainly have helped in assessing a possible involvement of the immune system in the reported reaction.

## 5. Conclusions

We reported a case of anaphylaxis secondary to the administration of SonoVue with a negative allergological evaluation. Given its widespread use and the potentially dangerous nature of the reactions it can cause, it is advisable to know how to promptly recognize potentially fatal reactions. In fact, we recommend safety measures such as the presence of qualified physicians who can quickly identify possible anaphylactic reactions, accessibility of adrenaline, and resuscitation equipment, in addition to the availability of performing serum tryptase level assay within 4 h, while performing these procedures. Further studies, however, need to be carried out to understand the mechanisms underlying the reactions caused by sulfur-hexafluoride-based contrast agents.

## Figures and Tables

**Table 1:** Characteristics of patients with severe hypersensitivity reactions after administration of SonoVue and Lumason reported in the literature.

Authors	Allergologic History	Known Diseases	Purpose	Type of Reaction	Therapy	Allergic Evaluation
Coudray et al. (2017) [7]	No	Hypertension	Control six months after endovascular abdominal aortic aneurysm repair (EVAR)	Anaphylaxis	Epinephrine + steroids + antihistamines + volume resuscitation + oxygen	Skin prick test for SonoVue and PEG were negative, serum histamine 88.9 nmol/L and acute serum tryptase 147 mcg/L
Geleijnse et al. (2009) [8]	Not reported	Hypertension, recent carotid stenting	Dobutamine stress echocardiography for risk stratification before aortic abdominal surgery.	Hypotension + skin erythema	Steroids + antihistamines + volume resuscitation	No
Geleijnse et al. (2009) [8]	Not reported	Pompe disease	To investigate cardiac function	Tinnitus, dizziness, hypotension, bradycardia	Steroids + antihistamines + volume resuscitation	No
Geleijnse et al. (2009) [8]	Not reported	Familial nonischemic dilated cardiomyopathy	To exclude non-compaction cardiomyopathy	Nausea, skin erythema, bradycardia and shock	Steroids + antihistamines + resuscitation procedure (cardiac massage and manual ventilation); after 10 h from discharge, the patient experienced pain in multiple joints lasting for 2 days	Type III allergic reaction without identification of a specific allergen
Ionescu (2009) [9]	No	Type-II diabetes, three-vessel coronary artery disease (angioplasty 6 weeks earlier)	Before elective dobutamine stress echo	Paresthesias, dizziness, bradycardia, hypotension, erythematous skin rash, tonic-clonic fit (after hypotension therapy), myocardial ischaemia	Atropine + steroids + antihistamines + midazolam + phenylephrine	No
Levano et al. (2012) [10]	No	Liver cirrhosis of unknown cause (Child–Pugh score B) and portal hypertension, esophageal varices	To evaluate a focal liver lesion	Anaphylaxis	Mechanical ventilation + fluids + steroids + antihistamines + norepinephrine	Skin prick and intradermal tests with SonoVue were negative, basal serum tryptase 3.74 mcg/L
Solivetti et al. (2012) [11]	No	Melanoma (surgically removed)	To evaluate a non-painful lesion in the periscapular region (suspected in-transit metastatic melanoma lesion)	Itching, heat sensation, edema of the face, respiratory difficulty, edema of the glottis, urticaria, cardiocirculatory collapse	Oxygen + fluids + steroids + antihistamines	No
Calvo et al. (2006) [12]	No	Acute anterior myocardial infarction and single vessel coronary disease	Elective dobutamine stress echo.	Sweating, bradycardia, hypotension and cardio-respiratory arrest	Cardiopulmonary resuscitation	No
Steinhauer et al. (2023) [13]	No	STEMI	To evaluate for left ventricular thrombus in the setting of apical akinesis	Dyspnea, loss of pulse, diffuse erythematous rash	Cardiopulmonary resuscitation, 2 mg IV epinephrine, 200 mg IV hydrocortisone, 50 mg IV diphenhydramine	Acute serum tryptase 23.9 mcg/L
Mikhail et al. (2023) [14]	No	Hypertension, atrial fibrillation, pericardial effusion, obstructive sleep apnea, diabetes mellitus type 2, asthma	To evaluate stroke	Shortness of breath, diaphoresis, cyanosis, hypoxia, tachycardia	Solumedrol, Albuterol, Diphenhydramine, and oxygen	No
Olson et al. (2018) [15]	No	Myelodysplastic syndrome, systemic mastocytosis	Ultrasound-guided biopsy of hepatic mass	Apnea and pulseless electrical activity	Epinephrine and cardiopulmonary resuscitation	Acute serum tryptase 72.4 mcg/L (serum basal tryptase was 41.5 mcg/L), leukotriene E4 6750 pg/mg Cr and N-methylhistamine 3897 mcg/g Cr
Mansour et al. (2022) [16]	No	Heart failure with preserved ejection fraction (HFpEF)	Echocardiogram to evaluate worsening dyspnea	Severe anaphylactic shock	Intravenous antihistamines, methylpredniso- lone and epinephrine	Not reported
Kerber et al. (2022) [17]	No	Not reported	Echo stress test	Nausea, vomiting, diarrhea, hypotension and hypoxia	Epinephrine, dexamethasone, benadryl, oxygen	Transient elevation in serum tryptase

## Data Availability

Dataset available on request from the authors.
